# Modulation of the ATP-Binding Cassette B1 Transporter by Neuro-Inflammatory Cytokines: Role in the Pathogenesis of Alzheimer's Disease

**DOI:** 10.3389/fphar.2018.00658

**Published:** 2018-06-20

**Authors:** Fawaz Alasmari, Charles R. Ashby, Frank S. Hall, Youssef Sari, Amit K. Tiwari

**Affiliations:** ^1^Department of Pharmacology and Toxicology, College of Pharmacy, King Saud University, Riyadh, Saudi Arabia; ^2^Department of Pharmacology and Experimental Therapeutics, College of Pharmacy and Pharmaceutical Sciences, University of Toledo, Toledo, OH, United States; ^3^Pharmaceutical Sciences, College of Pharmacy, St. John's University Queens, New York, NY, United States

**Keywords:** Alzheimer's disease, ABCB1, pro-inflammatory cytokines, amyloid beta, blood brain barrier

## Introduction

Inflammation of neuronal tissue, or neuro-inflammation, is associated with neurological diseases, including Alzheimer's disease (AD) (Patel et al., 2005; Walters et al., 2016; Wang B. et al., 2016). The exact role of neuro-inflammation in AD remains uncertain as it may be a result of other causative factors in AD, but can subsequently contribute to the course of the disease, or be caused by other factors. Neuro-inflammation is significantly correlated with changes in the expression of brain proteins that regulate the transport or signaling pathways of endogenous and exogenous molecules (Tilleux and Hermans, 2007; Kim et al., 2015; Gao et al., 2017). ATP-binding cassette (ABC) proteins, such as ABCB1 (P-glycoprotein, P-gp), are highly expressed in the brain capillary endothelial cells of the blood - brain barrier (BBB) and limit the uptake of certain endogenous and exogenous compounds into the brain (Löscher and Potschka, 2005; Zhang et al., 2015). Several studies have reported alterations in the expression and functions of ABCB1 in AD models (Wijesuriya et al., 2010; van Assema et al., 2012). The formation of amyloid beta (Aβ) (a substrate of ABCB1) plaques in the brain is a histological hallmark associated with AD (Lee et al., 2004; Wildburger et al., 2017). The ABCB1 transporter removes Aβ from the brain into the circulatory system (Hartz et al., 2010; ElAli and Rivest, 2013). Thus, alterations in the expression or function of ABCB1 may affect the progression of AD. The role of ABCB1 in AD progression and treatment has been recently reviewed, elsewhere (Pahnke et al., 2014; Sita et al., 2017). However, the focus of this opinion article is to discuss the effects of neuro-inflammatory cytokines on ABCB1 function and their role in the pathogenesis of AD.

## Alzheimer's disease is associated with neuro-inflammation

AD is the leading cause of dementia in the elderly and its prevalence has significantly increased over the last two decades (Reitz and Mayeux, 2014). Epidemiological studies indicated that more than 4.5 million people in the United States (U.S.) had AD in 2000 and this number may triple by 2050 (Hebert et al., 2003). Learning and memory impairments, as well as cognitive dysfunction, have been observed in animal models of AD (Dao et al., 2013; Webster et al., 2014; Xiao et al., 2017). Notably, AD is characterized by the formation of neurofibrillary tangles and amyloid beta (Aβ) plaques and the loss of cholinergic neurons in multiple brain regions (Paulson et al., 2008; Iba et al., 2013; Parent et al., 2013). The cleavage of amyloid precursor protein (APP) by the enzymes beta (β) secretase and gamma (γ) secretase produces Aβ in the brain (O'Brien and Wong, 2011). Studies indicate that Aβ causes neuro-inflammation through various signaling pathways (Liu et al., 2012; Parajuli et al., 2013). Aβ affects inflammatory signaling by activating toll-like receptor-2 (TLR-2) (Liu et al., 2012). Additionally, the incubation of human monocytes *in vitro* with Aβ (10 μM) for 30 min increases the mRNA expression of the pro-inflammatory cytokine IL-1β, while incubation for 48 h increases tumor necrosis factor-α (TNF-α) (Yates et al., 2000). This study also reported that concurrent incubation of mouse microglial cells with fibrillar Aβ (10 μM) and lipopolysaccharide (6.25, 12.5, or 25 ng/ml) for 48 h significantly increased the release of IL-1β and TNF-α compared to microglial cells that were incubated with only lipopolysaccharide. A prior study reported that nucleotide-binding and oligomerization domains, as well as caspase-1, are involved in oligomeric Aβ-induced interleukin-1β (IL-1β) processing (Parajuli et al., 2013). Further studies found that Aβ activates NLRP3 (nucleotide-binding domain, leucine-rich-containing family, pyrin domain-containing-3)/caspase1 inflammasome signaling pathway, resulting in neuro-inflammation induction (Gold and El Khoury, 2015; Saresella et al., 2016). The mRNA and protein expression of the NLRP3 inflammasome was increased in monocytes in individuals with moderate or severe AD (Saresella et al., 2016). The activation of this pathway has been found to increase the production of active inflammatory cytokines such as IL-1β (Gold and El Khoury, 2015). This indicates that stimulating the NLRP3/caspase1 inflammasome/IL-1β cascade might affect ABCB1 function or expression in AD patients. Studies are warranted to explore the pharmacological role of this pathway in modulating ABCB1 in AD models. In addition, IL-1β was detected in the nucleus basalis (NB) 24 h following the injection of Aβ (4 μg/μL) into the NB of rats (Giovannini et al., 2002). This effect was associated with activation of microglia and p38 MAPK pathway. Conversely, the incubation of cortical glial cells with IL-1 (100 ng/ mL) for 14 h or IL-6 (50–200 ng/mL) for 6 h significantly increased the mRNA expression of APP (Del Bo et al., 1995). Pro-inflammatory cytokines, such as TNF-α or interferon-γ (IFN-γ), have been reported to increase the production of Aβ in astrocytes expressing APP (Yamamoto et al., 2007). TNF-α, IFN-γ, and IL-β1 have been shown to stimulate γ-secretase, thereby increasing Aβ levels (Liao et al., 2004). Moreover, the pre-incubation of neuroblastoma cells with 0.1 mM of ibuprofen for 12 h significantly reduced Aβ secretion induced by 24 h of incubation with TNF-α and IFN-γ (Blasko et al., 2001). These data suggest that targeting these signaling pathways stimulated by Aβ could provide a pharmacological strategy to attenuate neuro-inflammation associated with AD, potentially improving AD symptoms and slowing disease progression.

## Role of neuro-inflammatory cytokines in neurodegenerative diseases and psychiatric diseases, and their effect on ABCB1 expression

The production of pro-inflammatory cytokines has been found in pre-clinical models of various neurodegenerative diseases. For example, transient focal ischemia was reported to be associated with an increase in the levels of TNF-α (Chu et al., 2007). Moreover, the concentrations of IL-1β, IFN-γ, and TNF-α are increased in the brains of animals following traumatic brain injury produced by Feeney's weight-drop model (Wei et al., 2012). Long-term exposure to drugs of abuse, such as ethanol, produces a significant increase in TNF-α concentrations in the hippocampus of rats (Alfonso-Loeches et al., 2010; Marshall et al., 2016b). The exposure of C57BL/6J mice and Wistar rats to ethanol significantly increases IL-1β mRNA expression and concentrations, respectively, in the brain compared to the control groups (Alfonso-Loeches et al., 2010; Marshall et al., 2016a). In addition, a significant increase in immunoreactive TNF-α in glial cells has been reported in the substantia nigra of Parkinson's disease (PD) patients compared to the control group (Boka et al., 1994). Furthermore, the levels of IL-1β and IL-6 are significantly increased in striatal dopaminergic neurons of PD patients (Mogi et al., 1994). IL-1β levels were increased in the frontal cortex and hippocampus of AD patients compared to individuals with vascular dementia and control subjects (Cacabelos et al., 1994). Importantly, the level of inflammatory cytokines was positively correlated with the level of Aβ in a mouse model of AD (Patel et al., 2005). These findings indicate that the levels of pro-inflammatory cytokines are increased in pre-clinical neurodegenerative and psychiatric diseases models, as well as the disease states they are purported to model.

Several studies investigated the effects of certain pro-inflammatory cytokines on the expression and function of ABCB1 (Evseenko et al., 2007; Iqbal et al., 2012; Walther et al., 2015). The mRNA and protein expression of *Abcb1*/ABCB1 were significantly decreased following incubation with TNF-α (30 ng/mL) for 24–72 h (Walther et al., 2015). Furthermore, incubation of cytotrophoblasts with TNF-α (20 ng/mL) or IL-1β (2 ng/mL) significantly decreased the expression of the *Abcb1*/ABCB1 mRNA and protein (Evseenko et al., 2007). ABCB1 function and mRNA levels in cultured guinea pig brain endothelial cells (harvested at postnatal day 14) were significantly decreased following incubation with TNF-α, IL-1β, or IL-6, at 3.3 × 10^3^ or 10^3^ pg/mL, for 24 h (Iqbal et al., 2012). Thus, neuro-inflammatory cytokines affect the expression and function of ABCB1, suggesting that neuro-inflammation in neurodegenerative diseases, including AD, may alter ABCB1 expression, although this remains to be determined.

## The impact of modulating ABCB1 on the progression of alzheimer's disease

Data suggest a relationship between neuro-inflammation, regulation of ABCB1 transporter, and Aβ clearance in the brain. The release of IL-1β, IL-6, and TNF-α are increased in brain micro-vessels compared to larger vessels of AD patients (Grammas and Ovase, 2001). Overall, IL-1 levels are also significantly increased in the brain of AD patients, compared to control subjects (Griffin et al., 1989). IL-6 levels in the cortex and hippocampus of AD patients are greater than those in control subjects (Bauer et al., 1991; Strauss et al., 1992). Since these cytokines, as discussed above, can regulate ABCB1 expression, an increase in pro-inflammatory cytokines in the brains of AD patients could reduce ABCB1 expression or function, contributing to the pathogenesis or progression of AD. A reduction in the expression of ABCB1 may lead to the accumulation of substances in the brain that promote inflammation or contribute to neurodegeneration in AD, most notably Aβ. Indeed, ABCB1 overexpression attenuated neurodegeneration in a mouse AD Model (Qosa et al., 2012; Durk et al., 2014), which is likely due to its transport of Aβ. In a mouse model of AD, increased Aβ is eliminated from the brain by ABCB1 (Bruckmann et al., 2017). St. John's Wort also significantly decreased the accumulation of Aβ in the brain, in part, by increasing the expression of the ABCB1 protein in mice (Brenn et al., 2014). These findings were further supported by studies reporting that 1α,25-dihydroxy-vitamin D3 significantly decreased the concentrations of Aβ in the cerebral cortex of mouse AD model (Durk et al., 2014) and increased ABCB1 activity and expression in brain capillaries of rats and mice, as well as in isolated endothelial cells of human micro-vessels (Chow et al., 2011; Durk et al., 2012). Rifampicin (20 mg/kg i.p., once daily for 3 weeks) had prophylactic efficacy against the development and progression of symptoms in a mouse AD model by decreasing Aβ levels via the upregulation of ABCB1 transporters in brain microvessels (Qosa et al., 2012). These findings were confirmed by previous studies showing that ABCB1 is critical in Aβ uptake across the BBB using ABCB1-knockout mice (Wang W. M. et al., 2016).

**Figure 1:**
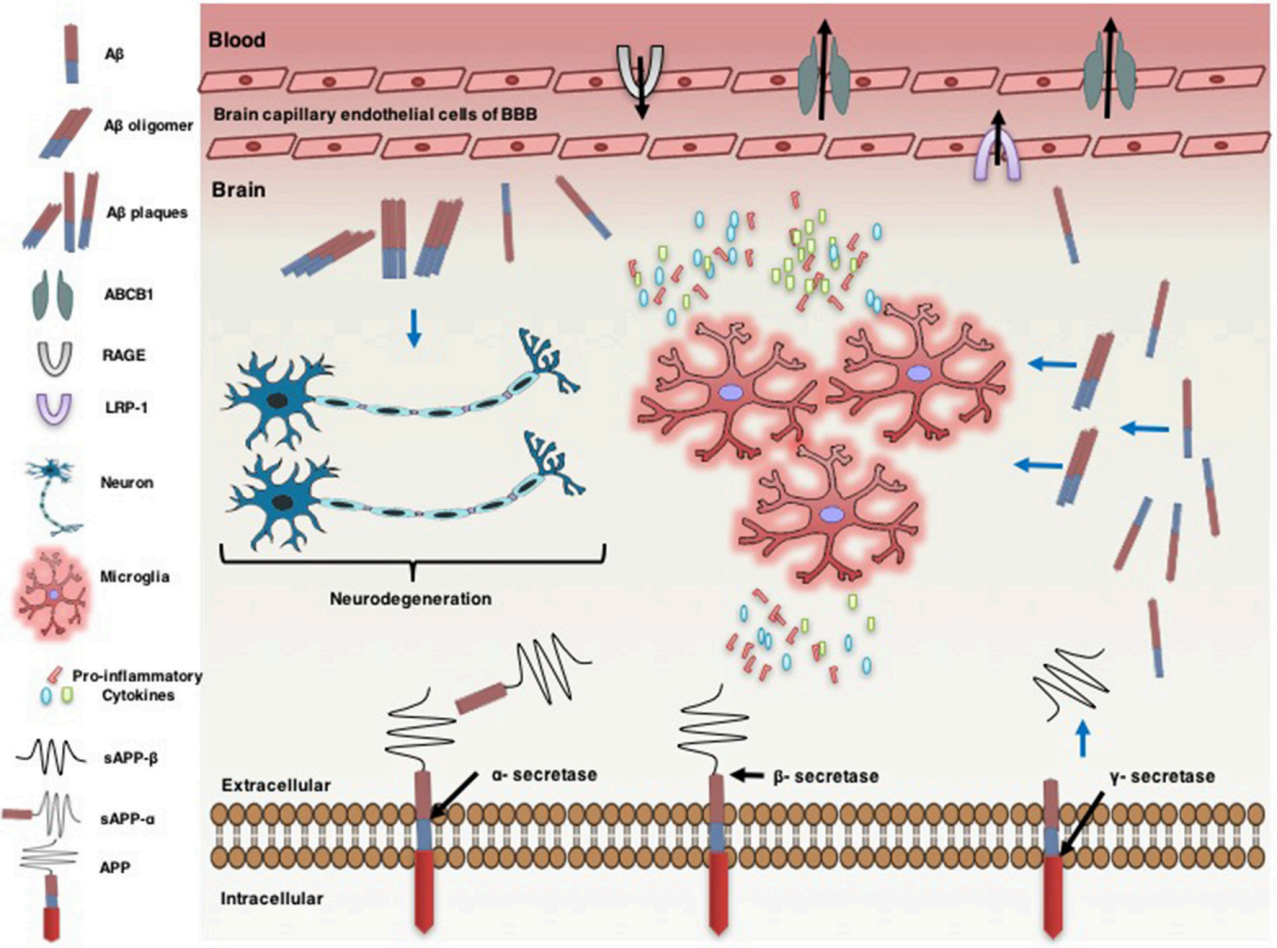
Neuro-inflammatory cytokines modulate ATP-binding cassette B1 (ABCB1), affecting the pathogenesis of Alzheimer's disease. Alzheimer's disease is associated with an increase in the formation of amyloid beta (Aβ) protein in the brain. Amyloid precursor protein (APP) generates Aβ through sequential proteolysis by beta (β) secretase and gamma (γ) secretase enzymes. Soluble APP-alpha (sAPP-α) and soluble APP-beta (sAPP-β) are produced through cleavage of APP by α-secretase and β-secretase, respectively. Aβ is transported across brain into the blood and vice versa by lipoprotein-related protein-1 (LRP-1) and the receptor for advanced glycation end products (RAGE), respectively. ABCB1 is also involved in uptake of Aβ from the brain into the circulatory system. However, the increase of Aβ plaque levels in the brain accelerates neuro-degenerations. The accumulation of Aβ leads to neuro-inflammation, characterized by activated microglia and the production of pro-inflammatory cytokines [e.g., interleukin-1β (IL-1β), IL-6, and tumor necrosis factor-α (TNF-α)]. Pro-inflammatory cytokines overexpression down regulates ABCB1 expression in the endothelial cells of brain capillary at the blood brain barrier (BBB). Pro-inflammatory cytokines increase the expression of APP. This effect is associated with a further increase in the accumulation of Aβ in the brain.

Thus, there are data indicating the involvement of the ABCB1 transporter in Aβ transport and targeting this transporter may attenuate the progression of AD. The treatment of mice with 5 mg/kg/day IP of oleocanthal, an anti-inflammatory compound, significantly increases the clearance of Aβ from the cerebral cortex of mice, in part, by upregulating ABCB1 transporters in brain micro-vessels (Qosa et al., 2015). This effect was associated with a decrease in IL-1β levels and a decrease in the activation of astrocytes. This indicates that neuro-inflammation may contribute to the accumulation of Aβ in the brain by decreasing the expression of ABCB1 transporters, which could contribute AD pathogenesis (Figure 1). Importantly, Aβ induced an increase in the release of pro-inflammatory cytokines (Yates et al., 2000; Liu et al., 2012; Parajuli et al., 2013), which may further reduce the expression of the ABCB1 transporter in a positive feedback loop that might contribute to the long-term trajectory of the illness. This hypothesis was supported by a previous study reporting that administration of Aβ-42 at 4 μg/h via subcutaneous transplanted ALZET pumps for 24 h significantly decreased the expression of *Abcb1* mRNA at the BBB of 90-day old mice (Brenn et al., 2011). A recent study reported that the incubation of isolated rat brain capillaries with Aβ-40 (10 nM for 6 h) significantly decreased the expression and transport activity of the ABCB1 transporter and this effect was associated with a degradation of the ubiquitin-proteasome (UP) (Hartz et al., 2016). The inhibition of UP in the microglial cell line enhanced the secretion of TNF-α (Kwon et al., 2008). Therefore, a decrease in the ABCB1 transporter expression in the BBB may lead to deposition of Aβ in the brain, contributing to the progression of AD.

## Conclusion and future directions

The accumulation of Aβ, a substrate of ABCB1, in the brain is associated with a decrease in the expression of ABCB1, which could affect pathogenesis of AD. We hypothesize that pro-inflammatory cytokines decrease the expression of ABCB1 in the endothelial cells of the BBB, reducing Aβ efflux, based on data from using preclinical models of AD. The upregulation of the ABCB1 transporter could decrease the accumulation of Aβ, thereby potentially attenuating the progression of AD. Future research is warranted to determine the precise role of neuro-inflammatory signaling pathways in regulating ABCB1 expression, and in the pathogenesis of AD, including cognitive effects. We suggest that compounds or treatments with dual actions, including anti-inflammatory actions and ABCB1 stimulatory effects, may have greater efficacy in reducing the progression of AD. Indeed, in a recent study, new compounds with dual actions for the treatment of AD symptoms were synthesized, although these compounds also targeted other pathways (Pang et al., 2017). The overexpression of ABCB1 may be pivotal in attenuating AD symptoms induced by the deposition of Aβ in the brain. Finally, reducing the accumulation of Aβ in the brain would also lead to a decrease in the levels of pro-inflammatory cytokines from microglia, limiting the down-regulatory effects of these cytokines on ABCB1, and interrupting the positive feedback loop between Aβ and neuro-inflammation that may be critical to disease progression in AD.

## Author contributions

FA, AT, and FH put in the idea and drafted the opinion letter. YS and CA helped provide valuable inputs and edited the opinion letter.

### Conflict of interest statement

The authors declare that the research was conducted in the absence of any commercial or financial relationships that could be construed as a potential conflict of interest.
